# 

**Published:** 1907-10-19

**Authors:** 


### BOOKS RECEIVED.

Henry Frowde and Hodder and Stoughton.

"A Manual of Venereal Diseases." By Officers of the R.A.M.C. 5s. net.

"Diseases of the Nose." By E. B. Waggett, M.A., M.B. 5s. net.

"Diseases of the Ear." By Hunter Tod, M.A., M.B. 5s. net.

Baillière, Tindall, and Cox.

"Marine Climates in Tuberculosis." By W. Ewart, M.D. 1s. net.

John Bale, Sons, and Danielsson, Ltd.

"Some of the Clinical Aspects of Pneumonia." By Donald W. C. Hood, C.V.O., M.D.

Cassell and Co., Ltd.

"Light and $x$-Ray Treatment of Skin Diseases." By Malcolm Morris, F.R.C.S., and S. Ernest Dore, M.D.

"Surgical Applied Anatomy." By Sir Fredk. Treves, Bart. 5th edition. Revised by Arthur Keith, M.D.

Health Resorts Bureau.

"Wintering in Rome." By A. G. Welsford, M.D.

Kegan, Paul, Trench, Trübner and Co., Ltd.

"A Spirit Message from Robert Burns."

Henry Kimpton.

"Blood Examination and Its Value in Tropical Diseases." By Claud F. Fothergill, M.R.C.S.

Macmillan and Bowes, Cambridge.

"Malaria." By W. H. S. Jones, M.A.

Smith, Elder and Co.

"First Aid to the Injured." Translated from the German by H.R.H. Princess Christian.

THE HOSPITAL *October* 19, 1907.

*Name* ..................................................................................

*Address* ..................................................................................

..................................................................................

This Coupon must accompany manuscript or contributions intended for The Hospital.

**B.P.C.** **JUST PUBLISHED.** **B.P.C.**

### THE BRITISH PHARMACEUTICAL CODEX

*Published by Authority of*

THE PHARMACEUTICAL SOCIETY OF GREAT BRITAIN.

This work, which has been in course of preparation for more than four years, is

**AN IMPERIAL DISPENSATORY**

for the use of Medical Practitioners and Pharmacists. The volume contains 1,434 medium 8vo. pages, and includes the most recent information respecting all drugs and medicines in common use throughout the British Empire, the descriptions of the various substances being supplemented by chemical and pharmacological notes, with suggestions as to

**THE BEST METHODS OF PRESCRIBING AND DISPENSING THE REMEDIES.**

The work is rendered especially valuable to medical practitioners by reason of the information it contains regarding the properties of drugs, and the conditions and diseases in which galenical preparations are usually given. The notes on the properties of drugs have been prepared under the guidance of Dr. W. E. Dixon, Professor of Pharmacology, King's College, London, and are not mere excerpts from medical literature, but original and concise descriptions of physiological action, which should assist medical practitioners to formulate a rational therapy and to construct their prescriptions in a scientific and practical manner.

**Price 12/6 net (abroad 13/6) delivered.**

*Orders, accompanied by remittances, may be sent direct to*

THE PUBLISHER, B.P.C., 72 Great Russell Street, W.C.

*NOW READY.*

**NERVE DISEASES FOR STUDENTS.**

By L. A. Clutterbuck M.D., B.S. (Durham), M.R.C.P. (London), L.R.C.P. & S. (Edin.), L.F.P. & S. (Glasg.), L.R.C.P. (Ireland); Hon. Physician, St. Marylebone General Dispensary; Clinical Assistant, West End Hospital for Nervous Diseases, London, &c.

Price 3s. net, by post 3s. 3d.

Of all Booksellers, or of the Publishers—
THE SCIENTIFIC PRESS, LTD.,
28/29 Southampton Street, Strand, London, W.C.

**PHARMACY, MATERIA MEDICA, AND THERAPEUTICS**

Is now rewritten and printed in large type, and is made a companion to the following volume. It contains an account of the action **of every drug in the B.P.,** as well as a section upon all the newest drugs, with an Introduction to the Art of Dispensing. With woodcuts, prescriptions, &c.

Also FOURTH EDITION. 1055 pages, 8vo. 16s.

**DICTIONARY OF TREATMENT.**

Containing an up-to-date Article upon the Treatment of every Diseased Condition, whether Medical, Surgical, Gynæcological, or Ophthalmological, as well as Articles upon Poisoning, Drowning, and the various Surgical Emergencies and Accidents, arranged for rapid Reference.

By Sir W. WHITLA, LL.D., M.D., Professor of Materia Medica, Queen's College, Belfast; Senior Physician Royal Victoria Hospital, &c.

The Publisher will supply both volumes carriage paid, for 21/-

London: HENRY RENSHAW, 356 Strand.

A LIST of the NEW PUBLICATIONS of the SCIENTIFIC PRESS, LIMITED, will be sent post free to any member of the Medical Profession on receipt of post card.

28 & 29 SOUTHAMPTON STREET, STRAND, LONDON, W.C.

